# Genomics-informed nursing strategies and health equity: A scoping review protocol

**DOI:** 10.1371/journal.pone.0295914

**Published:** 2023-12-15

**Authors:** Dzifa Dordunoo, Jacqueline Limoges, Patrick Chiu, Rebecca Puddester, Lindsay Carlsson, April Pike

**Affiliations:** 1 University of Victoria, School of Nursing, Director, Centre for Evidence informed Nursing and Health Care: JBI Centre of Excellence, Victoria, Canada; 2 Athabasca University, Chair, Ontario Cancer Research Ethics Board, Toronto, Canada; 3 Athabasca University, Edmonton, Canada; 4 Memorial University of Newfoundland, Faculty of Nursing, St. John’s, Canada; 5 Princess Margaret Centre, Toronto, Canada; Xiamen University - Malaysia Campus: Xiamen University - Malaysia, MALAYSIA

## Abstract

**Objective:**

The objective of this scoping review is to map the available evidence on strategies that nurses can use to facilitate genomics-informed healthcare to address health disparities.

**Introduction:**

Advancements in genomics over the last two decades have led to an increase in the delivery of genomics-informed health care. Although the integration of genomics into health care services continues to enhance patient outcomes, access to genomic technologies is not equitable, exacerbating existing health disparities amongst certain populations. As the largest portion of the health workforce, nurses play a critical role in the delivery of equitable genomics-informed care. However, little is known about how nurses can help address health disparities within the context of genomics-informed health care. A review of the literature will provide the necessary foundation to identify promising practices, policy, and knowledge gaps for further areas of inquiry.

**Inclusion criteria:**

We will include papers that explore strategies that nurses can undertake to facilitate genomics-informed care to address health disparities.

**Methods:**

This review will be conducted using JBI methodology for scoping reviews. We will search electronic databases including MEDLINE (OVID), EMBASE, Cochrane Library, PsychInfo, and CINAHL for quantitative and qualitative studies, systematic reviews and grey literature. Theses, books, and unavailable full-text papers will be excluded. The search will be limited to papers from 2013 and beyond. Two reviewers will screen titles and abstracts followed by full-text and disagreements will be resolved by a third reviewer. We will use a data extraction tool using Microsoft Excel and analyse data using descriptive statistics and conventional content analysis. Findings will be presented in the form of evidence tables and a narrative summary. We will report findings using the Preferred Reporting Items for Systematic reviews and Meta-Analyses extension for Scoping Reviews (PRISMA-ScR).

**Discussion:**

Genomics will continue to transform all aspects of health care across the wellness continuum from prevention, assessment, diagnosis, management, treatment, and palliative care. The identification of nursing strategies to address health disparities will build the foundation for policy and practice to ensure that the integration of genomic technologies benefits everyone.

## Introduction

Genomics is the study of genes within the genome to better understand how they interact with each other, the environment, and other psychosocial and cultural factors [1]. The promise of health and social improvements from genomic technologies has generated considerable enthusiasm and is revolutionizing health care across the healthcare continuum [2]. Advancements in genomics have also enabled precision health, which integrates genomic data with other relevant sources of information such as environmental, behavioral, and biological data to generate targeted healthcare strategies [3]. The diagnosis of rare diseases has improved, enabling tailored management and treatment options, and supporting preventive measures for individuals at-risk for genetic conditions. Common conditions such as cancer and cardiovascular disease have also been greatly impacted by earlier detection and new treatments derived from biomarker and genetic testing. Despite these significant gains, there is evidence that access to genetic testing and the subsequent benefits from the integration of genomics into healthcare are not evenly experienced [4–12]. In this way, genomic technologies are contributing to health disparities, in particular for people who have lower socioeconomic status, live in rural settings or who experience negative impacts of racial discrimination [4–12]. For example, sickle cell anemia is an inherited single point mutation on the HBB gene that affects the structure and functions of red blood cells [13]. It is common amongst people whose ancestors come from areas such as sub-Saharan Africa and India [13]. The institution of pre-conception and newborn screening would significantly reduce the burden of sickle cell anemia, yet this has not happened as 300 000 babies globally are born each year with it [13]. Genomic technologies should not widen existing health disparities and social disadvantage, but rather, contribute to an equitable and prosperous future for all individuals [14].

There are over 20 million nurses worldwide working in every sector of the health system and in all geographic regions of the world [15]. A central feature of nursing is to uphold equity and social justice in healthcare [16–21] and to build trusted partnerships with historically and systematically marginalized communities [22]. However, as it relates to genomics in most countries, there is limited infrastructure and support to develop evidence, policy, education, or research to guide the integration of genomics into nursing practice [23–25]. This is creating a situation where nurses cannot fully participate in interprofessional collaborative care, research, or use their full scope of practice in this genomic era. Moreover, this lack of guidance and coordinated activity could lead to nurses inadvertently perpetuating misinformation, myths, and stereotypes during care.

Jurisdictions such as the United States and United Kingdom have developed more robust infrastructure such as policy documents outlining scope of practice, standards, and competencies to guide nurses in providing genomics-informed health care. These policy documents outline the unique role that nurses have in patient assessment, diagnosis, care planning, education and counseling, and referral. They also support nurses to know how to address ethical, social, and legal issues associated with genomics-informed care and outline how they can contribute to health outcomes and health equity. The breadth and depth of nursing involvement in healthcare requires nurses to recognize the impacts of genomic technologies on widening health inequities and to be equipped with strategies to mitigate these harms. When equity and ethical issues associated with genomics are not addressed, the barriers to accessing precision healthcare are disproportionately experienced by the very segments of the society that need it the most (e.g., racial and ethnic minorities), exacerbating existing health disparities and resulting in poor health outcomes [5, 6, 9, 11, 12, 26].

Scholars have identified the need for infrastructure such as education, policy, research, and practice strategies to assist in countering biases and stereotypes of historically and systematically marginalized groups within the health system that contribute to health disparities [23, 26–29]. Further, many countries also have their own unique equity issues. For example, in Canada, there are unique equity and health disparity issues tied to access to genetic testing and genomics, particularly amongst various Indigenous Peoples, that must be understood and addressed by all health care professionals [23]. Identifying helpful nursing practices that address health disparities can provide clarity for those providing education or creating policy to guide practice and research.

Scholarship from jurisdictions such as United States, Canada, China, and Turkey have explored the knowledge, attitudes, confidence, and practices in genomics amongst nurses and show that in general, nurses have low to moderate levels of genomic knowledge. These studies show that nurses are interested in learning more about genetics and genomics; however, there is limited infrastructure and support to assist them to acquire the needed knowledge, skills, and competencies [23–25, 30–34]. A critical feature of infrastructure is specific guidance on strategies that can help address the equity concerns arising from the integration of genomics into health care delivery. As a result, to accelerate the integration of genomics into nursing practice, we need to identify the most promising nursing-specific practices and policies that will facilitate safe and equitable interprofessional collaborative care and identify knowledge gaps that require research and activities to enable the integration of genomics and precision healthcare for the benefit of all.

A preliminary search of PROSPERO and MEDLINE indicated that there have been no systematic reviews or scoping reviews completed, and there are no current or reviews underway on nursing strategies to facilitate genomics-informed health care to address health disparities. By synthesizing the existing literature on nursing strategies that facilitate genomics-informed healthcare to address health disparities, meaningful knowledge can be generated. We chose to conduct a scoping review as this methodology is well suited for examining emerging evidence when it is still unclear [35]. Ensuring that practice, education, and policy are developed in ways that assist nurses to mitigate the dynamics of privilege and marginalization is crucial to the safe and equitable integration of genomics. Further, it will help us create and share an action and research agenda, including policy recommendations to address equity issues associated with genomics-informed nursing practice. Thus, the purpose of this scoping review is to map the available evidence on strategies that can be used by nurses to facilitate genomics-informed healthcare to address health disparities.

## Review question

The research question guiding this scoping review is: what is the available evidence on how nurses can facilitate genomics-informed healthcare to address health disparities?

### Eligibility criteria

#### Population

The population of interest includes nurses of all designations including registered nurses, registered/licensed practical nurses (sometimes referred to as enrolled nurses in some jurisdictions), registered psychiatric nurses, and nurse practitioners. Nurses in all domains of practice and specialty areas will be included. In jurisdictions where the role of the nurse and midwife is interrelated (e.g., nurse-midwife), these papers will be included as the focus of this scoping review is on examining the breadth and depth of strategies that nurses used to facilitate genomics-informed healthcare to address disparities. Given that nursing scope of practice is broad and there may be overlap with other health professions, papers that discuss all health care professionals including nurses (with no disaggregated data) will be included to ensure all strategies are captured.

#### Concept

The key concept of interest is strategies that address health disparities. We define strategies as any methods, processes, interventions, and tools used by nurses to address and mitigate health disparities in the provision of genomics-informed healthcare (e.g., providing education, collecting and interpreting a family health history, referrals). We define health disparities as the “systematic differences in health status or in the distribution of health resources between different population groups, arising from the social conditions in which people are born, grow, live, work and age” [36]. For example, cancer disproportionately impacts those with lower socioeconomic status, people living in rural Canada, and Indigenous peoples [37, 38]. Similarly, research has shown ethnic, racial, and geographic disparities in cardiovascular health that are tied to access to genomics-informed healthcare [39]. Advances in genomics and precision healthcare have significant implications for minimizing the burden of diseases through earlier detection, faster diagnostics, and targeted treatment [40–44].

#### Context

The review will include all relevant global literature from all geographic locations that examine strategies that can enable nurses to engage in genomics-informed health care to address health disparities. Nurses work in various settings and contexts within the health system which positions them to be change agents in the adoption of genomics-informed health care [3]. For example, nurses working in maternal child health and with new families can draw on knowledge of genomics to understand how early life experience affects health and diseases over the lifespan and across generations [45, 46]. Those working in oncology can support patients in the process of diagnostic, detection, and treatment of cancer. Nurses who are equipped with genomic-informed health care strategies can assist people to address modifiable factors such as diet, exercise, and stress, which can influence gene expression. Ultimately, this enables nurses to support patients to reduce their risks through education, prevention, screening, and support for lifestyle changes to ensure everyone can experience positive outcomes associated with genomics.

### Types of sources

We will consider experimental and quasi-experimental study designs including randomized controlled trials, non-randomized controlled trials, before and after studies and interrupted time-series studies. In addition, analytical observational studies including prospective and retrospective cohort studies, case-control studies and analytical cross-sectional studies will be considered for inclusion. This review will also consider descriptive observational study designs including case series, individual case reports and descriptive cross-sectional studies for inclusion. Qualitative studies will be considered including, but not limited to, designs such as phenomenology, grounded theory, ethnography, qualitative description, interpretive description, action research and feminist research. In addition, systematic reviews that meet the inclusion criteria will be considered, depending on the research question. Text and opinion papers as well as grey literature will be considered for inclusion in this scoping review. We will search for grey literature using international nursing genomics organizations including the International Society of Nursing in Genomics (ISONG) and the Global Genomics Nursing Alliance (G2NA). Books will be excluded given the lack of methodological guidance on how to include these sources in scoping reviews; theses will be excluded as they are not peer-reviewed; and unavailable full text articles will be excluded as they will not be retrievable. See S1 Appendix for inclusion and exclusion criteria.

## Methods

We will conduct this scoping review in accordance with the JBI methodology for scoping reviews [47]. This methodology is based on Arksey and O’Malley’s [48] initial framework which has been further enhanced by Levac et al. [49] and Peters et al. [47]. The review will be organized into six stages: 1) identifying the research question and aligning it with the review objectives; 2) identifying relevant studies using a inclusion and exclusion criteria that is aligned with the research objective and questions; 3) selecting relevant studies using a planned approach to evidence searching, selection, data extraction, and the presentation of evidence; 4) charting the data using both descriptive statistics and qualitative content analysis; 5) consulting an advisory committee; and 6) collating, summarizing, and reporting the evidence.

### Search strategy

The search strategy will aim to locate both published and unpublished studies. A research librarian will be consulted throughout the development of the search strategy and protocol. An initial limited search of MEDLINE (OVID) was undertaken to identify articles on the topic (see S2 Appendix). The text words contained in the titles and abstracts of relevant articles, and the index terms used to describe the articles will be used to develop a full search. The search strategy, including all identified keywords and index terms, will be adapted for each included database and/or information source. The reference list of all included sources of evidence will be screened for additional studies. Papers that are not in English will be translated using artificial intelligence (AI) technology. Papers published within the last ten years (since 2013) will be included given the focus on seeking the most up-to-date evidence in accordance with grant funding requirements. Databases to be searched include MEDLINE (OVID), EMBASE, Cochrane Library, PsychInfo, and CINAHL. Due to the focus on global literature, we will search for unpublished studies and grey literature from international genomics nursing organizations including the International Society of Nurses in Genetics (ISONG) and the Global Genomics Nursing Alliance (G2NA).

### Study/source of evidence selection

Following the search, all identified citations will be collated and uploaded into Covidence (2023) and duplicates removed. Covidence will be used as it enables collaboration amongst multiple reviewers. Following a pilot test using 10% of the studies, titles and abstracts will then be screened by two or more independent reviewers for assessment against the inclusion criteria for the review. Potentially relevant sources will be retrieved in full text. The full text of selected citations will be assessed in detail against the inclusion criteria by two or more independent reviewers. Reasons for exclusion of sources of evidence at full text that do not meet the inclusion criteria will be recorded and reported in the scoping review. Any disagreements that arise between the reviewers at each stage of the selection process will be resolved by a third reviewer. Citations of included studies will be uploaded into Mendeley, a reference manager software program. The results of the search and the study inclusion process will be reported in full in the final scoping review and presented in a Preferred Reporting Items for Systematic Reviews and Meta-analyses extension for scoping review (PRISMA-ScR) flow diagram [50].

### Data extraction

Data will be extracted from papers included in the scoping review by two independent reviewers using a data extraction tool developed by the reviewers using Microsoft Excel (2019). The data extracted will include specific details about the population (nurses), concept (genomics-informed nursing strategies), context (all geographic locations and studies focused on health disparities), study methods and key findings relevant to the review question. A draft extraction form is provided (see S3 Appendix). The draft data extraction tool will be modified and revised as necessary during the process of data extraction. We will pilot the extraction form using 10% of the included articles and resolve any discrepancies through discussion. Modifications will be detailed in the scoping review. If appropriate and when required, authors of papers will be contacted to request missing or additional data. Critical appraisal of individual sources of evidence will not be completed as this is generally not required for scoping reviews.

### Data analysis and presentation

We will organize results using evidence tables to ensure data are presented in a clear and structured format. We will use descriptive statistics to illustrate frequency counts and percentages for data such as year of publication, type of nurses discussed, geographic region, type of publication, and categories developed through content analysis. We will use conventional content analysis [51] to identify equity issues and genomics-informed nursing strategies to address health disparities. Conventional content analysis is an appropriate analytical method as it enables the development of broader categories based on coded data. A narrative summary will accompany the evidence tables and describe how the results relate to the review objective and question. To ensure the overall reporting quality of this scoping review, reporting will follow the Preferred Reporting Items for Systematic reviews and Meta-Analyses extension for Scoping Reviews (PRISMA-ScR) [50]. All retrieved records from the search strategy, included and excluded records from primary and secondary screening, and records retrieved from other sources will be reported using an adapted PRISMA flow diagram to enhance the reproducibility of this scoping review.

## Discussion

Health equity is an essential component of the quintuple aim of health care alongside enhanced patient and provider satisfaction, population health outcomes, and cost-effectiveness [52]. Genomics will continue to transform health care and improve population health; thus the introduction of new technologies should equally benefit all peoples and groups within society. As the largest portion of the health workforce, nurses can play a leading role in identifying health disparities and require evidence-informed practices to engage in collaborative interdisciplinary care that promotes equity. Our review will enable the nursing workforce to understand the nature, extent, and range of nursing strategies that already exist to address health disparities within the context of genomics-informed care and will identify gaps that need to be addressed through policy and practice development. Given our broad geographical focus, findings from the review will enable us to explore the different contexts in which strategies have been implemented and/or evaluated, and the considerations required to adopt them in diverse settings.

## Supporting information

S1 Appendix Inclusion/exclusion screening form. (DOCX)

S2 Appendix MEDLINE (OVID) search strategy. (DOCX)

S3 Appendix Data extraction form. (DOCX)
