# Supplementary material for: Genomics-informed nursing strategies and health equity: A scoping review protocol
Source: PLoS One. 2023 Dec 15;18(12):e0295914. doi: 10.1371/journal.pone.0295914 (PMC10723661; doi:10.1371/journal.pone.0295914)
Supplement: S1 Appendix — (DOCX) [file pone.0295914.s001.docx]

**Appendix A: Inclusion/Exclusion Screening Form**

| **Criteria** | **Process** |
| --- | --- |
| 1. **Language** | The full text is available in English or can be translated into English via AI technology   - If no, exclude based on language - If yes, move to criteria 2 |
| 1. **Sources** | The paper is either published primary research (quantitative, qualitative, mixed methods, systematic reviews, text and opinion papers, or grey literature (exclude theses and books)   - If no, exclude based on source - If yes, move to criteria 3 |
| 1. **Geographical location** | Papers from all geographic regions will be included.   - Move to criteria 4 |
| 1. **Population** | The paper focuses on nurses (registered nurses, registered psychiatric nurses, licensed practical nurse, nurse practitioners, nurse midwives) or health care professionals including nurses   - If no, exclude based on population - If yes, move to criteria 5 |
| 1. **Concept** | The paper focuses on genomics-informed nursing strategies   - If no, exclude based on concept - If yes, move to criteria 6 |
| 1. **Context** | The paper focuses on health disparities / health inequities   - If no, exclude based on context - If yes, include paper |
