# Supplementary material for: Genomics-informed nursing strategies and health equity: A scoping review protocol
Source: PLoS One. 2023 Dec 15;18(12):e0295914. doi: 10.1371/journal.pone.0295914 (PMC10723661; doi:10.1371/journal.pone.0295914)
Supplement: S2 Appendix — (DOCX) [file pone.0295914.s002.docx]

**Appendix B: MEDLINE (OVID) Search Strategy**

| **#** | **Query** | **Results from 25 May 2023** |
| --- | --- | --- |
| 1 | nurs*.tw,kf. | 530,217 |
| 2 | ("genomics informed health care" or "genomics informed healthcare" or "genomics informed" or genomic* or genetic* or hereditary or "family history" or "family health" or "precision health" or "precision medicine" or "cascade screening" or "cascade testing").tw,kf. | 1,613,278 |
| 3 | (genetic* adj5 (test* or screen* or service*)).tw,kf. | 72,688 |
| 4 | (equalit* or disparit* or inequalit* or equit* or inequit*).tw,kf. | 196,210 |
| 5 | ((Health or healthcare or "health care") adj5 (disparit* or inequit* or equalit* or status or equit* or inequalit*)).tw,kf. | 155,710 |
| 6 | Nurse's Role/ or exp Nurses/ | 131,940 |
| 7 | Genomics/ or Genetic Testing/ | 107,609 |
| 8 | Healthcare Disparities/ or Health Status Disparities/ or Health Equity/ | 41,202 |
| 9 | 1 or 6 | 577,938 |
| 10 | 2 or 3 or 7 | 1,638,692 |
| 11 | 4 or 5 or 8 | 297,101 |
| 12 | 9 and 10 and 11 | 199 |
| 13 | limit 12 to yr="2013 -Current" | 123 |
