# Supplementary material for: Genomics-informed nursing strategies and health equity: A scoping review protocol
Source: PLoS One. 2023 Dec 15;18(12):e0295914. doi: 10.1371/journal.pone.0295914 (PMC10723661; doi:10.1371/journal.pone.0295914)
Supplement: S3 Appendix — (DOCX) [file pone.0295914.s003.docx]

**Appendix C: Data Extraction Form**

| **Field** | **Findings** |
| --- | --- |
| **Reference ID** |  |
| **Author** |  |
| **Year** |  |
| **Geographic region of study/paper**   - Country |  |
| **Type of publication**   - Quantitative - Qualitative - Text and opinion papers - Systematic review - Grey literature |  |
| **Purpose or aim of study** |  |
| **Equity issue discussed including**   - Type of health disparity - Root causes - Outcomes |  |
| **Population**   - Registered Nurses - Licensed Practical Nurse - Registered Psychiatric Nurses - Nurse Practitioners - Nurse Midwives |  |
| **Disease process / conditions** | E.g., for hereditary cancer (e.g., HBOC, Lynch), Hypercholesterolemia |
| **Genomics-informed strategies to address health disparities** | E.g., education, family health history, referrals |
| **Domain of strategies**   - Clinical practice - Education - Leadership |  |
